# Early Detection of Pre-Cancerous and Cancerous Cells Using Raman Spectroscopy-Based Machine Learning

**DOI:** 10.3390/cells12141909

**Published:** 2023-07-21

**Authors:** Uraib Sharaha, Daniel Hania, Itshak Lapidot, Ahmad Salman, Mahmoud Huleihel

**Affiliations:** 1 Department of Microbiology, Immunology, and Genetics, Faculty of Health Sciences, Ben-Gurion University of the Negev, Beer-Sheva 84105, Israel; sharaha@post.bgu.ac.il; 2 Department of Biology, Science and Technology College, Hebron University, Hebron P760, Palestine; 3 Department of Green Engineering, SCE—Shamoon College of Engineering, Beer-Sheva 84100, Israel; dan308103@gmail.com; 4 Department of Electrical and Electronics Engineering, ACLP-Afeka Center for Language Processing, Afeka Tel-Aviv Academic College of Engineering, Tel-Aviv 69107, Israel; itshakl@afeka.ac.il; 5 Laboratoire Informatique d’Avignon (LIA), Avignon Université, 339 Chemin des Meinajaries, 84000 Avignon, France; 6 Department of Physics, SCE—Shamoon College of Engineering, Beer-Sheva 84100, Israel

**Keywords:** cancer, Raman spectroscopy, machine learning, normal fibroblast cells (NFC), NIH/3T3, MBM (cancerous cells)

## Abstract

Cancer is the most common and fatal disease around the globe, with an estimated 19 million newly diagnosed patients and approximately 10 million deaths annually. Patients with cancer struggle daily due to difficult treatments, pain, and financial and social difficulties. Detecting the disease in its early stages is critical in increasing the likelihood of recovery and reducing the financial burden on the patient and society. Currently used methods for the diagnosis of cancer are time-consuming, producing discomfort and anxiety for patients and significant medical waste. The main goal of this study is to evaluate the potential of Raman spectroscopy-based machine learning for the identification and characterization of precancerous and cancerous cells. As a representative model, normal mouse primary fibroblast cells (NFC) as healthy cells; a mouse fibroblast cell line (NIH/3T3), as precancerous cells; and fully malignant mouse fibroblasts (MBM-T) as cancerous cells were used. Raman spectra were measured from three different sites of each of the 457 investigated cells and analyzed by principal component analysis (PCA) and linear discriminant analysis (LDA). Our results showed that it was possible to distinguish between the normal and abnormal (precancerous and cancerous) cells with a success rate of 93.1%; this value was 93.7% when distinguishing between normal and precancerous cells and 80.2% between precancerous and cancerous cells. Moreover, there was no influence of the measurement site on the differentiation between the different examined biological systems.

## 1. Introduction

Cancer comprises a growing health, economic, and social issue [1], accounting for nearly 10 million deaths in 2020, or nearly one in six deaths. For many years, studies of the incidence and relevance of cancer have informed us of the outcomes for tumors detected at an advanced stage, prompting research into methods to identify the disease before symptoms appear [2]. Cancer causes the transformation of normal cells into tumor cells in a multi-stage process that generally develops from a precancerous lesion to a malignant tumor.

These changes result from the interaction between a person’s genetic factors and three categories of external agents—physical, chemical, and biological carcinogens [3]. Malignant cells are characterized, among other factors, by the acceleration of the cell cycle, genomic alterations, invasive growth, increased cell mobility, and changes in the cellular surface. Through its alterations, the nucleus of a cancerous cell contributes significantly to the evaluation of the tumor malignancy. Its surface, volume, nucleus/cytoplasm ratio, shape, density, structure, and homogeneity are all changed [4]. Nucleus segmentation; invaginations; changes in chromatin, such as a decrease in heterochromatin or an increase in interchromatin and perichromatin granules; an increase in nuclear membrane pores; and the creation of inclusions are all connected to the ultrastructural properties.

Moreover, the cytoplasm also experiences changes; either new structures are created or typical structures disappear. The cytoplasm becomes basophilic due to its ribosomal and messenger RNA buildup. The cytoplasm of malignant cells is sparse and usually contains vacuoles. In addition, all cell organelles undergo dramatic changes, including mitochondria, the Golgi apparatus, the granular endoplasmic reticulum, peroxisomes, and lysosomes. For example, mitochondria show high variability in shape and volume, and huge mitochondria can sometimes be observed, while the Golgi apparatus in malignant cells is generally poorly developed [4]. In line with the fact that cancerous cells undergo many changes compared with normal cells, these changes include all sites of the cell, nucleus, cytoplasm, and membrane. Age-related risk accumulation for certain malignancies is the most likely cause of the dramatic increase in cancer incidence.

The successful treatment of cancer depends not only on effective therapies but also on improved methods to assess an individual’s risk of developing cancer and the ability to detect cancer at early stages. In addition to the typical risk increase, aging individuals tend to have less efficient cellular repair systems. Thus, most diagnosed cancer cases occur in individuals aged 65 or older [5,6]. The early detection of cancer can shorten the treatment period, increase the likelihood of recovery, and minimize patients’ and their families’ financial and personal costs and the costs to society as a whole [7]. Cancer patients undergo a long period of testing until precancerous or cancerous cells are discovered. Currently, three steps of testing are used to diagnose cancer. The first assessment is often a physical screening test or laboratory tests such as urine and blood tests. The second test includes a series of imaging tests, which can include computerized tomography (CT) scans, bone scans, magnetic resonance imaging (MRI), positron emission tomography (PET) scans, ultrasound, and X-ray. The third test is usually a biopsy, which is subjected to staining and histological analysis [8,9]. Most of the tests are time-consuming and produce discomfort and anxiety for patients. While they wait for the interpretation of each test result, the tumor continues to develop in the body and may require more extensive treatment. All the above illustrate the need for a fast, reliable, and accurate method for the determination of cancerous cells at the initial stages of their development.

Raman spectroscopy is known as a reliable, sensitive, and accurate method that can identify and characterize biochemical changes in cells at the molecular level, which accompany abnormal development, including cancer. Raman spectroscopy, or Raman scattering, is based on the interaction between a laser beam and a sample and enables the measurement of the vibrational energy of the tested sample. Thus, the set of Raman shifts that correspond to the vibrational energies are recorded, and they are called the Raman spectrum, which is considered a “biochemical fingerprint” of the tested sample [10,11,12,13]. The combination of Raman spectroscopy and machine learning has become a powerful diagnostic tool that has shown great potential in the medical field in recent years [14]. Previous research has demonstrated that Raman spectroscopy might be used to characterize and identify a variety of biological samples. These include the identification of biochemical changes due to the proliferation of mammalian cells in culture [15], the detection and characterization of cells infected with herpes viruses [16,17], the identification of spectral features that characterize human coronary atherosclerosis [18], and the identification of different subtypes of cancer. These cancer subtypes include cervical cancer [19,20], melanoma [21,22], breast cancer [23,24,25], lung cancer [26], colorectal cancer [27], and others. In our previous study, we examined the potential of Raman spectroscopy for the identification and characterization of murine fibroblast cell lines (NIH/3T3) and malignant mouse fibroblast cells and achieved promising results, with a success rate of approximately 87.0% when the measurements were taken from the cell membrane [27]. In this study, we resumed our previous work, and Raman spectroscopy was used for the identification of cells at the early stages of cancer development by measuring three systems: primary mouse fibroblast cells, which represented normal and healthy cells (NFC); a mouse fibroblast cell line (NIH/3T3), which represented precancerous cells; and fully malignant mouse fibroblasts (MBM-T). The cells of the three biological systems were measured from three different areas of the cell (cell center, cytoplasm, and cell membrane) to identify the area responsible for the main spectral differences between the three models. This aimed to improve our previous results mainly through discrimination between primary and precancerous cells, enabling us to monitor the changes that occur in the cells at very early stages of malignant cell transformation. Undoubtedly, this subject is very important and cancer is considered one of the most important issues within society; therefore, more studies are required to achieve the main goal, which is the early and rapid detection of cancer, so that it can be treated, controlled, and coexisted with.

## 2. Materials and Methods

The main phases of the current research were (a) treatment and cell growth; (b) preparation of the samples for Raman measurements; (c) acquisition of the Raman spectra; (d) preprocessing of the spectra and feature extraction; and (e) analysis of the data using LDA and the decision system.

### 2.1. Cells

Three different cell types, NFC, NIH/3T3, and MBM-T, were investigated to follow the development of cancer. These cells represented the normal, precancerous, and cancer categories, respectively.

The NFC cells were fibroblast cells of mice embryos with a short life span [28], making it necessary to re-establish these cultures frequently.

The NIH/3T3 (murine fibroblast cells) were precancerous cells that had undergone many changes due to mutations throughout various transfers; however, such cells are not considered cancerous cells because they do not have all the properties of a cancerous cell [10]. These cells are defined as abnormal cells because they may undergo further changes over time that will convert them into cancerous cells [29].

The MBM-T cells were fully malignant mouse fibroblasts with all the characteristics of cancer cells [30].

The three types of cells were grown in sterile flasks at 37 °C, with RMPI-1640 medium (Biological Industries, Kibbutz Beit-Haemek, Israel), containing 10% fetal bovine serum (FBS) (Gibco, Billings, MT, USA), 2 mM fetal bovine glutamine (Biological Industries, Kibbutz Beit-Haemek, Israel), and 100 µg/mL of an antibiotic, penicillin (Biological Industries, Kibbutz Beit-Haemek, Israel). The cell cultures were incubated at 37 °C with 5% CO_2_.

### 2.2. Sample Preparation for Raman Measurements

A quartz slide was wrapped with smooth aluminum foil and soaked with highly concentrated acetone (99.5%) as a substrate. All the cells were collected from the flasks using trypsin (Biological Industries, Kibbutz Beit-Haemek, Israel), and then transferred to an Eppendorf tube. The cells were then centrifuged at 240× *g* for five minutes. The cell pellet was washed three times with 500–1000 µL of NaCl buffer (0.9%). A hematocytometer determined the number of cells. Then, the cells were pelleted and re-suspended to achieve a cell concentration of 30–50 cells/µL. A drop of suspended cells was mounted on the substrate and dried before performing the Raman measurements.

### 2.3. Raman Measurements

Dried samples of representative cells were measured using the single spectrum mode in a Horiba LabRAM HR Evolution Raman Microscope with a Sincerity CCD detector that was deep-cooled to −60 °C, 1024 × 256 pixels. The basic microscope and detector were fitted with a 10 mW 532 nm illumination Nd:YAG green laser (with a laser spot size of 2 μm) and a 10% transmittance filter fixed at the probe laser station to avoid sample heating by the laser. An integration time of 60 s was used for all Raman measurements. The laser was focused by a 50× objective lens (Olympus MPLAN (Tokyo, Japan)) to generate a diffraction-limited spot size of 1.54 μm on the sample. A 600 L/mm grating was used to generate spectra with 1.2 cm^−1^ dispersion to maximize the signal strength while minimizing the background signal from auto-fluorescence. Wavenumber calibration was performed using a silicon sample.

**Figure 1:**
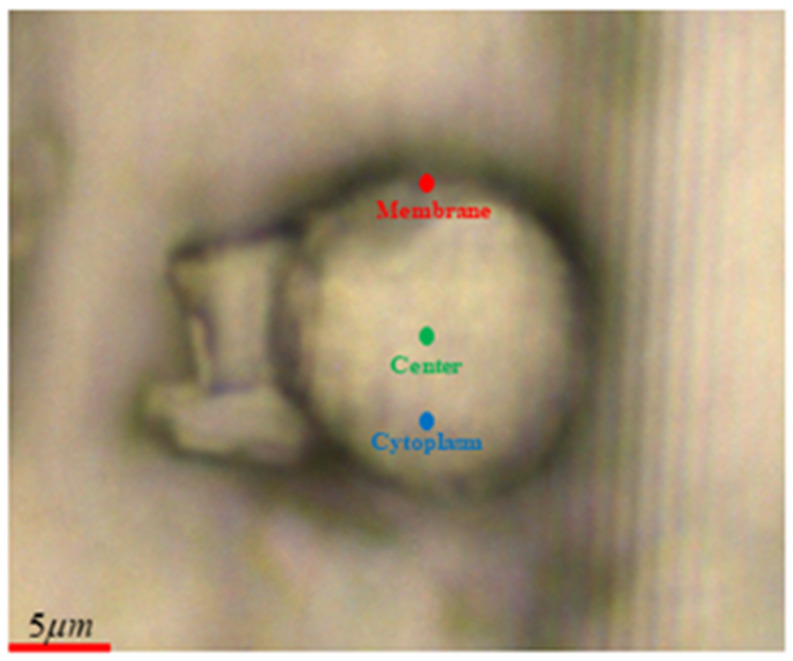
Typical NIH/3T3 cell, recognized using a Raman microscope with 100× magnification power. The three sites, center, cytoplasm, and membrane, are labeled.

The cells were prepared from different cultures and were measured for more than one year using the Raman facility. Moreover, three measurements were recorded for each cell, as detailed in Table 1. The measurements were performed on three different sites of each cell, namely the cell center, cell cytoplasm, and cell membrane, as shown in Figure 1.

**Figure 2:**
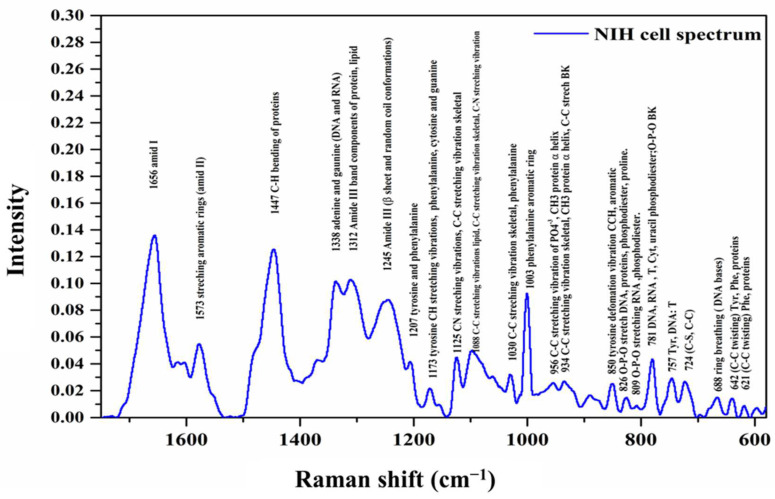
Raman spectrum of normal fibroblast cell measured in the 1800–600 cm^−1^ region. Characteristic Raman peaks have been labeled.

A typical Raman shift spectrum for a normal fibroblast cell is shown in Figure 2.

Table 2 lists all the prominent bands based on the published literature [31,32,33,34,35,36,37,38]. Each peak in the Raman shift spectrum is characteristic of a certain type of vibration of the functional groups of the main molecules, providing us with a detailed molecular fingerprint.

### 2.4. Spectral Preprocessing

To improve the quality of the spectra and Raman shift bands and to compare the different spectra, all the received spectra were preprocessed before the machine learning classification analysis. All preprocessing steps were performed using our in-house code (written in Python).

In the first step, the spectra were cut to the 1800–600 cm^−1^ range and smoothed using the Savitsky–Golay algorithm with 5 points, aiming to reduce the instrumental noise (of the device) and improve the spectral information.

In the next step, baseline correction was performed to eliminate the differences between the spectra resulting from fluorescence and spectral shifts in the baseline. The process was carried out in several stages [39]; the full (1800–600 cm^−1^) region was subdivided into two sub-regions, 1800–1201 cm^−1^ and 1200–600 cm^−1^.

The minima at fixed wavenumbers 1800 cm^−1^, 1750 cm^−1^, 1720 cm^−1^, 1560 cm^−1^, 1530 cm^−1^, and 1490 cm^−1^ were calculated in the first sub-region. The second sub-region was divided into 50 equal ranges, and the minimum at each range was calculated. In the next step, the minima points were connected using straight lines to create the baseline and subtracted from the spectrum; five iterations of the baseline correction process were performed.

The last step in the preprocessing process involved normalizing the spectra using vector normalization. The Raman intensities at all the measured wavenumbers were averaged and subtracted from the original spectrum. The resulting spectrum was treated as a vector and its “norm” was normalized to 1 by calculating the sum of the squares of all the Y values and dividing the spectrum by the square root of this sum. As a result of the first step (subtracting the intensities’ average from the original spectrum), some of the intensities of the normalized spectrum (Y-axis) were negative. Therefore, all spectra after vector normalization were corrected by shifting the minimum intensities to zero.

### 2.5. Machine Learning Analysis

The NFC, NIH/3T3, and MBM-T cells were treated as different categories; we aimed to predict the correct category by analyzing the Raman spectrum. PCA and LDA were used for the goals analysis to identify each category’s characteristic features [40,41,42]. In addition, the decision system was designed to use a classifier (biological system type: normal, precancerous, and cancerous) based on a feature vector to determine the category of the model.

### 2.6. Principal Component Analysis

The PCA technique is most often used for dimensionality reduction. In the current work, we used PCA for both data visualization and feature extraction. The eigenvectors of the covariance matrix are the principal components (PCs). The PCs with the highest eigenvalues capture a major part of the data variance. The projection of each measurement (in our case, the Raman spectrum) onto the PC gives a weight that indicates the contribution of this PC to the measurement. The weights of the PCs with the highest eigenvalues are a low-dimensional representation of the measurement [40].

### 2.7. Linear Discriminant Analysis

All the classification tasks in this study were binary. After the features were extracted by PCA dimensionality reduction, the LDA classifier was applied. In the case of uniform priors, LDA classification is performed according to the minimum of the Mahalanobis distance between the feature vector and the mean vector of each class [43]. The application of LDA provides class separability by constructing a linear decision region between the different classes.

In general, the classifier calculates an average for each group under the assumption of a Gaussian distribution with a shared covariance matrix; if the difference is only in the averages of each group, the separation is obtained by a hyper-plane (k − 1). When the data are two-dimensional (k = 2), the hyperplane is a straight line. When the data are three-dimensional vectors (k = 3), the hyperplane is a plane. This separation boundary serves as a decision system that determines the class of a certain feature vector [16,44].

### 2.8. Validation

Cross-validation using the K-fold approach was applied to evaluate the classification performance. Using K-fold, the database is partitioned into K groups. In this study, since the database was relatively small, five-fold cross-validation was used (K = 5). In this method, one fold is left for the test and the remaining four folds are used to train the classifier; thus, the training subset and the test subset are disjointed. In such a way, the predictive power of the LDA was evaluated.

To evaluate the statistical accuracy, the validation process was performed five times, and, in each repetition, a different fold was used for prediction. The performance of the binary LDA classifier was averaged and summarized in the confusion matrix, as illustrated in Table 3.

The deviation in the accuracy of the classifier was calculated as the standard deviation of the LDA classifier in each fold.

When the classification was performed between the normal category (NFC) and the abnormal category (combined NIH/3T3 and MBM-T), the abnormal category was determined as the positive state. When the classification was performed between couples of the three categories, the cancerous cells (MBM-T) were determined as the positive state in NFC-(MBM-T) and NIH/3T3-(MBM-T). When the classification was performed between NIH/3T3-NFC, the precancerous (NIH/3T3) cells were determined as the positive state.

Four statistical indices were obtained, as shown in Table 3: one true positive (TP), one false negative (FN), one false positive (FP), and one true negative (TN). TP describes the number of cases correctly predicted by the classifier as the positive group. FP describes the number of cases incorrectly predicted by the classifier as the positive group. TN describes the number of cases correctly predicted by the classifier as the negative group. FN describes the number of cases incorrectly predicted by the classifier as the negative group. The performance of the classifier was calculated in terms of the accuracy (ACC), sensitivity (SE), specificity (SP), positive expected value (PPV), and negative predictive value (NPV), according to Equations (1)–(5):

(1) $$ACC=\frac{TP+TN}{TP+TN+FP+FN}$$

(2) $$SE=\frac{TP}{TP+FN}$$

(3) $$SP=\frac{TN}{TN+FP}$$

(4) $$PPV=\frac{TP}{TP+FP}$$

(5) $$NPV=\frac{TN}{TN+FN}$$

## 3. Results and Discussion

The potential of Raman spectroscopy to distinguish between three biological systems—primary normal cells, precancerous cells, and cancerous cells—was investigated during the initial stage of this project. The analyses were based on 997 Raman spectra acquired from 457 different cells and cultures that were grown on different days, as detailed in Table 1.

**Figure 3:**
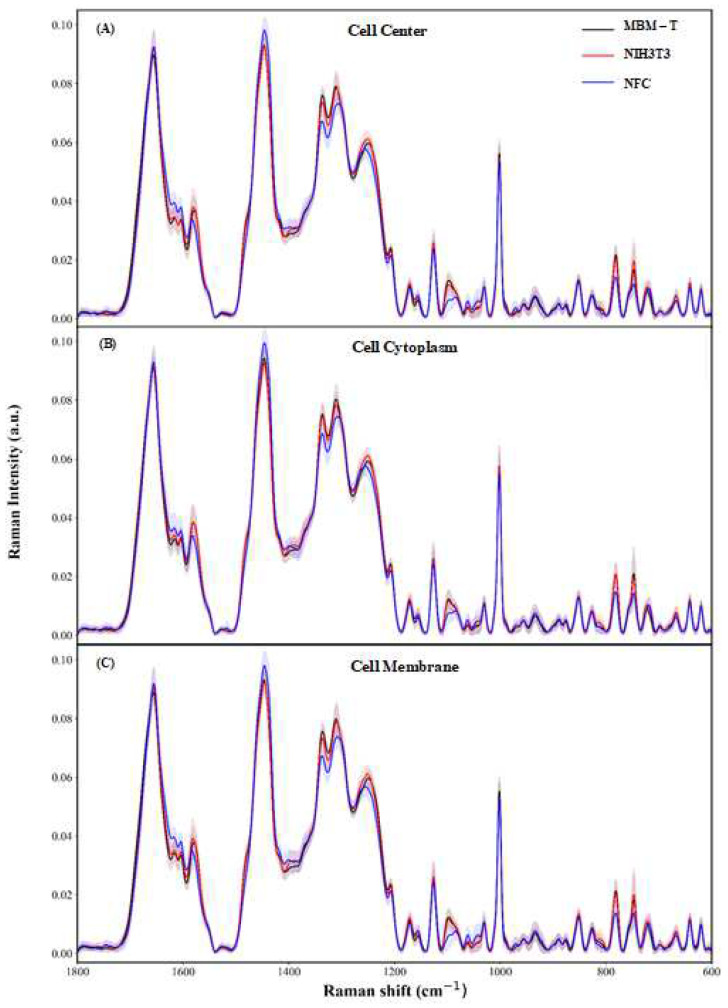
Averages of Raman spectra of NFC, NIH, and MBM-T cells in the 1800–600 cm^−1^ range after preprocessing, measured from different regions: (**A**) cell center, (**B**) cytoplasm, and (**C**) cell membrane. The errors were calculated as standard deviations and are shown as shadowed areas.

As mentioned, many changes in the components of all cells occur during the initiation of cancer. It is interesting to identify in which region of the cell the biochemical changes are dominant. With this goal, the spectra acquired from the different cell regions—cell center, cytoplasm, or cell membrane—were analyzed separately. Spectral differences correlate with the biochemical changes; thus, large spectral differences will lead to higher classification rates. Thus, the analyses were performed based on the spectra acquired from the different sites separately; the performance of the classifiers was compared. For the 1800–600 cm^−1^ region, Figure 3 displays the average spectra of the measurements from the different regions of each of the three biological systems investigated in the study: NFC, NIH/3T3, and MBM-T. As can be seen, the spectral changes were minor, making it impossible to distinguish between these biological systems using simple methods such as visual comparison.

**Figure 4:**
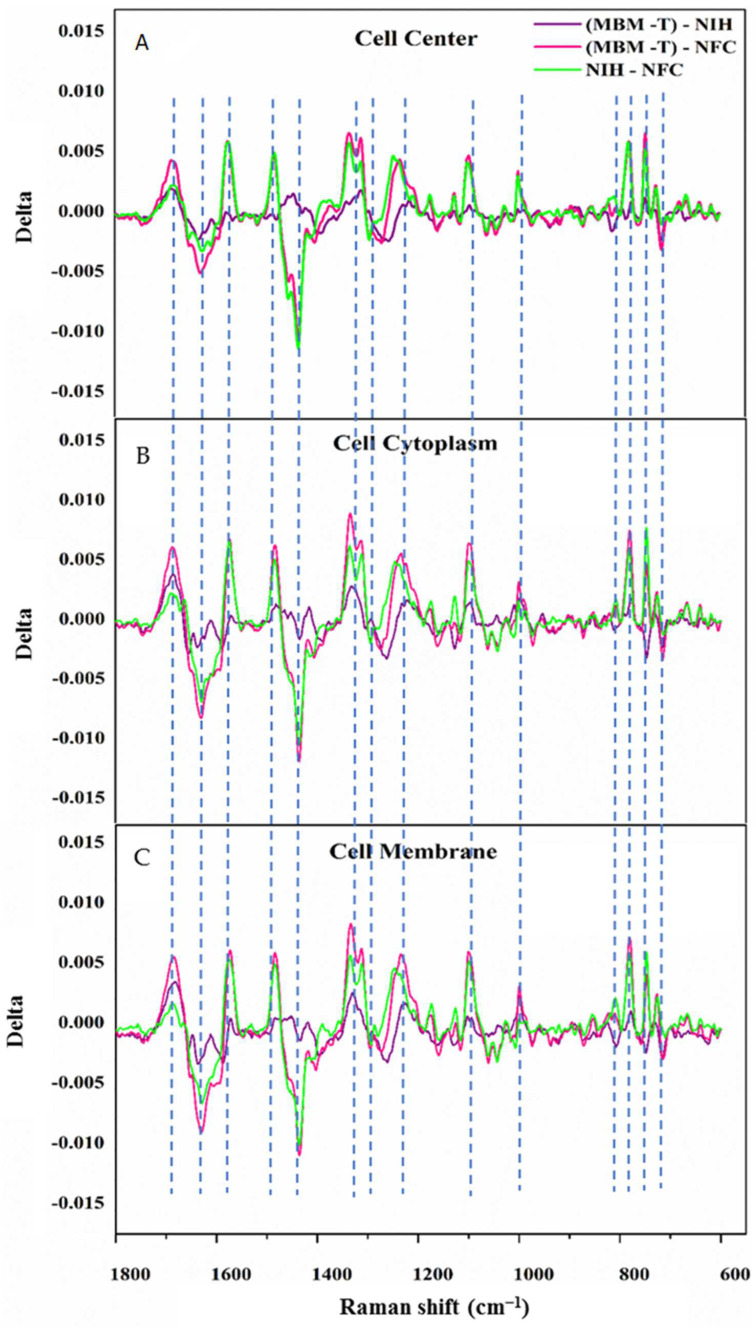
The differences in the average Raman spectra of (MBM-T)-NIH (purple), (MBM-T)-NFC (pink), and NIH-NFC (green) in the 1800–600 cm^−1^ wavenumber range. The Raman measurements were recorded from three different regions in the cell: (**A**) center region, (**B**) cytoplasm region, and (**C**) membrane region.

In particular, regarding these spectral Raman differences, the delta between each couple of the three systems was applied for each region (i.e., the cell center, cell cytoplasm, and the cell membrane), as shown in Figure 4. Figure 4 shows that the most noticeable differences between the average spectra of the three systems occurred in the wavenumber range of 700–800 cm^−1^, with a peak at 1064 cm^−1^; in the wavenumber range of 1096–1088 cm^−1^; at 1128 cm^−1^, 1250 cm^−1^, 1311 cm^−1^, 1337 cm^−1^, 1443 cm^−1^, and 1578 cm^−1^; and finally in the wavenumber range of 1602–1618 cm^−1^ and at 1700 cm^−1^, which arose from different components and likely also the structures of the main molecules that composed the biological samples (e.g., the proteins, fats, carbohydrates, and nucleic acids).

As is clear from Figure 4, the spectral differences between the MBM-T cells, representing cancer cells, and the NFC cells, representing normal cells, were the highest, followed by the spectral differences between NFC and NIH/3T3 cells, which represented precancerous cells.

However, the differences between the NIH/3T3 and MBM-T cells were the lowest and were considered very small. It is worth noting that these differences were prominent in different parts of the cell and were not specifically focused on one part of the cell. These results align with the biological hypothesis of the existence of significant changes in cells when they transform from normal cells to cancerous ones, including changes in the cell membrane, cell cytoplasm, and cell nucleus [45].

As is known, the development of a tumor and the host’s responses to it might be affected by the surface molecular alterations brought about by malignization, including changes in proteins and carbohydrates that function as enzymes and cell surface receptors [46]. Changes also occur in the cytoplasm, which are reflected by new proteins and other components [46]. Malignant cells have a small amount of cytoplasm, which frequently contains vacuoles [4].

Moreover, the cell organelles, which are usually distributed inside the cells in the cytoplasm and cell center, including the nucleus, undergo several changes.

Through its alterations, the nucleus of a cancerous cell contributes significantly to the evaluation of a tumor malignancy, as mentioned in the Introduction.

In addition, the granular endoplasmic reticulum appears to be a more streamlined structure and the cisternae may be clogged with amorphous, granular, or filamentous debris. Along with a decrease in the granular endoplasmic reticulum and an increase in free ribosomes and polysomes, tumor cells also exhibit an increase in free ribosomes and polysomes, which indicates that more proteins are being produced throughout the cell growth process [4].

**Figure 5:**
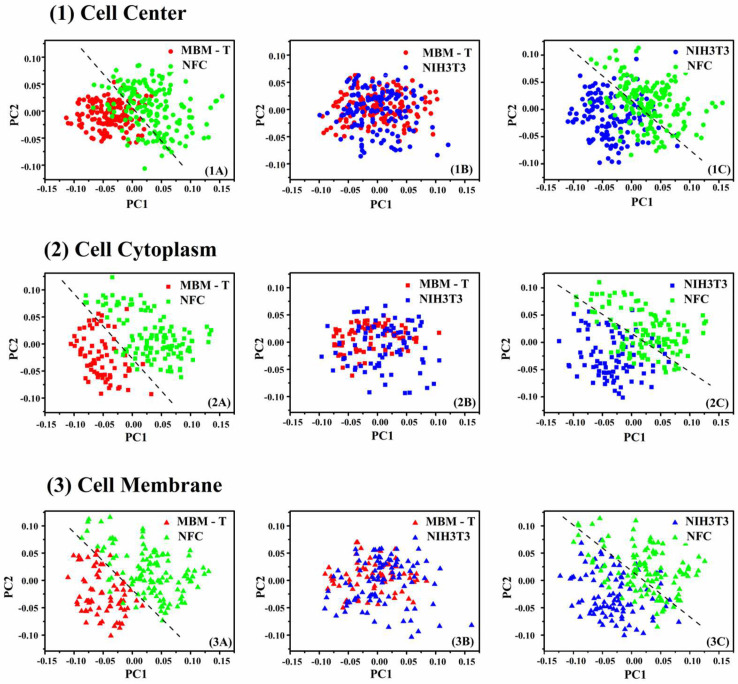
Two-dimensional plots of the PC score data projected onto the PC1–PC2 subspace for the comparison between the different pairs MBM-T-NFC, MBM-T-NIH/3T3, and NIH/3T3-NFC. Each spectrum is represented as one point in these plots, whose coordinates are the coefficients of the PCs (loadings) calculated by PCA. The red, blue, and green points represent the MBM-T, NIH/3T3, and NFC cells, respectively. The PCA calculation was based on Raman spectra measured from the different cellular regions: center (Line 1), cytoplasm (Line 2), and membrane (Line 3).

PCA projections of the data into a two-dimensional subspace were applied as a first step in the differentiation between the three tested biological systems (Figure 5). These plots were generated for visualization and to estimate the complexity of the classification problem. Moreover, these plots offered a clearer and more accurate understanding of the differences between the three biological systems, MBM-T, NIH-3T3, and NFC, while demonstrating the ability to distinguish between the three biological systems as pairs, based on the Raman spectra obtained from various cell regions. In Figure 5, two clear clusters can be seen in all the pairs of (MBM-T)-NFC and NIH/3T3-NFC, regardless of the region of measurement in the cell. Although there are two distinct clusters in each figure, there is still some overlap between the points; the overlap between the points is almost complete when the biological systems considered are precancerous and cancerous cells. Thus, the classification complexity of the precancerous and cancerous classes was higher compared to the classification between normal and cancerous cells and between normal and precancerous cells. Different projections onto different PC subspaces were examined and the best projections were found in the PC1–PC2 subspace, as presented in Figure 5.

Following the PCA analysis, the LDA classifier was applied for the classification of the three different biological systems, the MBM-T, NIH/3T3, and NFC biological systems, with four databases. In the first database (database I), the characteristic vector of each cell was the average of three Raman spectra measured from the center, cytoplasm, and cell membrane. Meanwhile, in the second database (database II), third database (database III), and fourth database (database IV), the characteristic vector of each cell was the Raman spectrum measured from the center, cytoplasm, and membrane of the cell respectively.

At this level, PCA was used for dimensionality reduction; thus, the feature vectors were the coefficients of the PCs. The eigenvectors for the projection were calculated for the training set only. The required number of PCs is task-dependent: when the differences between the classes are large, a small number of PCs is required (simple classification), while, when the differences between the classes are minor, a large number of PCs is required (hard classification). Thus, when classification was performed between each of the couples, namely abnormal (cancerous and precancerous)–normal, cancerous–normal, and precancerous–normal, 2–6 PCs were sufficient. When the classification was performed between the cancerous and precancerous categories, 10–20 PCs were required to enable the LDA classifier to achieve the best classification.

**Figure 6:**
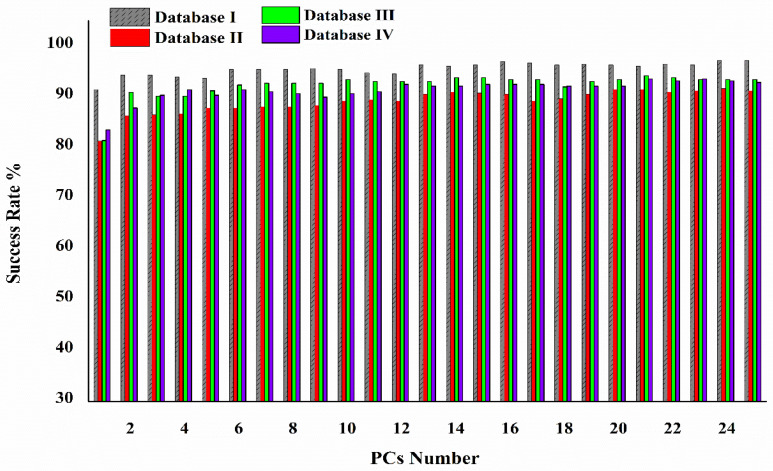
Success rate of the LDA classifier versus PC number for classification of normal and abnormal cells using the four databases.

The classifier’s performance was evaluated according to its classification success rate (Acc) regarding the number of PCs for the classification of normal and abnormal cells for the first 25 PCs. As illustrated in Figure 6, we created a graph of the success rate as a function of the number of PCs using databases I, II, III, and IV.

After a certain PC number, the accuracy rate reached a plateau, which means that increasing the number of PCs did not affect the LDA accuracy rate. The smallest number of PCs that enabled the classifier to reach the plateau was chosen.

Table 4 shows the classifier’s performance when classifying two different categories. The results show that Raman spectroscopy has the potential to detect any changes in cells that are likely to develop into cancer cells, with 93.1% Acc (Table 4a).

Moreover, Table 4b shows the classifier’s performance in classifying the precancerous cells NIH/3T3 versus cancer cells MBM-T, with 80.2% success. In addition, Table 4c,d show the binary LDA classifier’s performance in categorizing normal NFC cells and precancerous cells (NIH/3T3), normal NFC cells, and cancer cells (MBM-T), respectively.

From the performance of the classifier, presented in Table 4, it is evident that the classification of normal NFC cells and precancerous NIH/3T3 cells, and the classification of normal NFC cells and cancerous MBM-T cells, were the most successful for the database of average cells (database I), with 93.7% and 96.5% success, respectively. Additionally, although the classification of the measurements taken from the cytoplasm (database III) yielded slightly better results compared to the other measurement locations for both categories (c and d), the success rate of the classification was very high when taken from the three tested regions of the cell. These results are not surprising because, when normal cells are transformed, all the cell components, including the nucleus, cytoplasm, organelles, and membrane, undergo many changes. Changes cannot take place in one area without changes also taking place in others. Therefore, the average for all areas of each cell already includes most of the changes in the cell biomolecules during their transformation.

The cytoplasm region contains many proteins, organelles, and cell structures, so measurement from the cytoplasm can include changes in any of these structures, which might explain the slight superiority achieved when using this area for the classification of normal NFC cells and aberrant cells (NIH/3T3 and MBM-T).

The Raman spectrum is rich in features arising from both the chemical and morphological structures of various cells’ biomolecules, and due to their modes of vibration. Dividing the spectrum into sub-ranges may help to indicate which range and biomolecules have been significantly altered during cancer development. Moreover, it can help to determine the most informative range within the Raman spectra that can be used for discrimination between normal NFC and precancerous NIH/3T3 cells; consequently, it can be particularly useful for the early detection of cancer. The spectra were divided into four domains that characterized the main functional groups of the cells’ biomolecules. The range 1195–600 cm^−1^ was contributed mainly by carbohydrates and the 1380–1196 cm^−1^ range encompassed mainly proteins and lipids, while the 1520–1381 cm^−1^ range was mainly contributed by nucleic acids. In addition, the 1728–1521 cm^−1^ range encompassed mainly proteins (amide I and amide II). Table 5 shows the classifier’s performance for the classification of the three systems as pairs of normal NFC versus aberrant cells (NIH/3T3 and MBM-T), precancerous NIH/3T3 versus cancerous cells MBM-T, NFC versus NIH/3T3, and NFC versus MBM-T, in the four different ranges of the Raman spectra. The feature vectors in the LDA classifier of each cell were the average of three Raman spectra measured from the center, cytoplasm, and cell membrane (database I), which offered the highest success rate for differentiation.

Table 5 shows that the 1195–600 region yielded slightly greater differentiation success than other spectrum ranges in discriminating between NFC, NIH/3T3, and MBM-T; NFC and NIH/3T3; and NFC and MBM-T. In contrast, the 1380–1196 cm^−1^ range, which represents mostly proteins and lipids, showed greater success when discriminating between NIH/3T3 and MBM-T. Cancer cells typically have altered energy metabolism, including increased resting energy consumption and increased sugar, lipid, and protein metabolism [47]. Aerobic glycolysis is dominant in cancer cells, which means that even when oxygen is present, cancer cells mostly obtain their energy through glycolysis (the Warburg effect) [48].

Because cancer cells need a significant amount of energy to thrive, this produces significantly less energy than oxidative phosphorylation, which appears counterintuitive. However, the Warburg effect may benefit cancer cells because it provides precursors for many biosynthetic pathways, including amino acid precursors and NADPH and ribose sugars for DNA and RNA synthesis. Glycolytic enzymes such as GLUT1, lactate dehydrogenase, pyruvate kinase, and the lactate exporter are unregulated in cancer cells, whereas pyruvate dehydrogenase is inhibited, increasing glycolytic flux and reducing pyruvate’s ability to enter oxidative phosphorylation [48].

These facts regarding the metabolism of sugars in cancer cells present a clear and logical justification of the results, which indicated that the 1195–600 cm^−1^ region, contributed mainly by carbohydrates, had a greater ability to distinguish between normal and cancerous cells and between normal and precancerous cells. At the same time, the situation was different regarding the ability to distinguish between cancerous and precancerous cells, because both of them altered their energy metabolism. However, it is important to note that when using the entire spectrum (1800–600 cm^−1^), which included all changes in all cell biomolecules (proteins, lipids, nucleic acids, and carbohydrates), we were able to differentiate between cells more successfully. This was true despite the changes in the carbohydrate region being consistent with scientific facts.

As mentioned, all the cells of the three biological systems were measured from three different sites in the cell, the center, cytoplasm, and cell membrane, proving that there was no influence of the measurement site concerning the classification success rate. Moreover, because the spatial resolution of Raman spectroscopy is high (~1.5 µm), some of the spectral differences were due to the screening of different organelles during measurements.

It is preferable to take several measurements from different sites in the cell and use the average of the obtained spectra as a representative spectrum in the classification analyses, to reduce the spectral variation due to the screening of different organelles.

## 4. Conclusions

Raman spectroscopy is a potential method of discrimination between normal and precancerous or cancer cells and it can help in the early detection of cancer. A clear impact on the lives and health of patients, as well as the medical and economic fields more generally, will result from the ability to identify precancerous cells. Discrimination between normal and precancerous or cancerous cells is not related to specific regions of cells. It is recommended to take measurements from multiple locations in the cell and use the average of these measurements for differentiation. In addition, the use of the complete spectrum range (1800–600 cm^−1^), without segmenting it into separate ranges based on the primary biomolecule contributor, yields superior results than the use of one range alone.

## Figures and Tables

**Table 1:** Details of the number of cells and spectra that were acquired with the Raman spectrometer from the three different measured sites.

		NFC	NIH/3T3	MBMT	Total
No. of measurements	Cell center	169	132	143	444
	Cytoplasm	115	86	73	274
	Rich in membrane	116	91	72	279
Total No. of measurements		400	309	288	997
No. of cells		169	136	152	457

**Table 2:** Characterization and assignment of the peaks in the Raman spectrum.

Peak Position/cm^−1^	Major Assignments
1745	vw υ(C=O) lipid
1657	vs Amide I
1618	s υ(C=C) Tyr, Trp
1602	s υ(C=C) Phe, Tyr
1578	s DNA: A, G, C=C, δ(N–H) and υ(C–N) amide II
1528	vw–C=C–carotenoid
1443	vs δ(CH_2_) lipids, υ(C–H) proteins (collagen)
1398	s δ(CH_2_), C=O symmetric stretching
1337	s CH_3_CH_2_ γ of collagen and polynucleotide chain (DNA bases)
1311	s CH_3_CH_2_ τ of lipids, collagen, Trp
1250	s Amide III
1209	m υ(C–C_6_H_5_), Trp, Phe, Tyr
1175	w υ(C–H) Tyr, Phe, Cyt, G
1156	w υ (C–C, C–N) proteins, carotenoids
1128	m υ (C–N, C–C) Skeletal
1096	w symmetric υ(PO_2_^−^) DNA BK, υ(C–N)
1088	w υ(C–C) skeletal, υ(C–N) proteins, υ(C–C) lipid
1064	vw Proline (assignment to collagen)
1045	vw Collagen
1032	w υ(C–H) Phe, ν(C–C) skeletal, υ(C–N) proteins
1001	s υ(C–C), Symmetric ring breathing mode of Phe
971	vw Cyt (DNA and RNA)
957	w υ(C–C) hydroxyapatite, carotenoid, cholesterol, υ(PO_4_^−3^), (CH_3_) proteins (α-helix)
934	w υ(C–C) skeletal, proline, valine, protein BK (α-helix conformation), glycogen
889	w BK, proteins C–C skeletal
874	w υ(C–C) Hydroxyproline, Trp
850	w Ring breathing mode of Tyr, υ(C–C) proline ring
826	w υ(O–P–O) DNA, proteins, phosphodiester, proline, hydroxyproline, Tyr
811	vw phosphodiester, υ(O–P–O) RNA
781	m υ(O–P–O) DNA, RNA, T, Cyt, U, phosphodiester, BK
750	m Symmetric breathing of Trp, υ(C–S) Cys, DNA: T
721	w υ(C–S, C–C) protein, CH_2_ rocking, C–N (membrane phospholipid head)
668	w υ(C–S) Cys
642	w υ(C–C) τ Tyr, υ(C–S) protein, Phe
619	w υ(C–C) τ Phe, protein

s—strong, m—medium, w—weak, v—very, sh—shoulder, δ—deformation vibration, υ—stretching vibration, γ—wagging, τ—twisting, A—adenine, G—guanine, T—thymine, Cyt—cytosine, U—uracil, Glu—glutamic acid, Phe—phenylalanine, Trp—tryptophan, Tyr—tyrosine, Cys—cysteine, Met—methionine, BK—backbone.

**Table 3:** Typical confusion matrix of a binary classifier obtained after validation.

		Predicted	
		Positive	Negative
TRUE	Positive	True Positive (TP)	False Negative (FN)
	Negative	False Positive (FP)	True Negative (TN)

**Table 4:** Performance of the LDA classifier in distinguishing healthy NFC cells from aberrant cells (NIH/3T3 and MBM-T) in (**a**). The distinction between cancerous MBM-T cells and precancerous NIH/3T3 cells is shown in (**b**). The distinction between normal NFC cells and precancerous NIH/3T3 cells is shown in (**c**). The distinction between normal NFC cells and cancerous MBM-T cells is shown in (**d**). Four (I, II, III, and IV) databases were classified, where the feature vectors were the Raman spectra after dimensionality reduction using PCA.

**(a) NFC versus (NIH/3T3 + MBM-T)**								
**Database**	**No. of NFC**	**No. of Abnormal**	**No. of PCs**	**Acc (%)**	**SE (%)**	**SP (%)**	**PPV (%)**	**NPV (%)**
I	169	288	2	93.1 ± 3.2	97	96.9	94.8	98.2
II	169	275	4	88.4 ± 3.6	87	94.2	90.2	92.2
III	115	159	6	92.2 ± 3.1	93	95	93	95
IV	116	163	4	92.5 ± 2.9	89.7	96.3	94.5	92.9
**(b) NIH/3T3 versus MBM-T**								
**Database**	**No. of NIH/3T3**	**No. of MBM-T**	**No. of PCs**	**Acc (%)**	**SE (%)**	**SP (%)**	**PPV (%)**	**NPV (%)**
I	136	152	13	81.7 ± 3.3	77.9	85.5	82.8	81.3
II	132	143	20	80.2 ± 2.9	78	83.2	81.1	80.4
III	86	73	10	79.9 ± 2.5	74.4	86.3	86.5	74.1
IV	91	72	10	80.2 ± 2.1	82.5	80.6	81.4	79.5
**(c) NFC versus NIH/3T3**								
**Database**	**No. of NFC**	**No. of NIH/3T3**	**No. of PCs**	**Acc (%)**	**SE (%)**	**SP (%)**	**PPV (%)**	**NPV (%)**
I	169	136	3	93.7 ± 3.6	98.2	92.6	94.3	97.7
II	169	132	5	87.4 ± 3.4	92.9	84.8	88.7	90.3
III	115	86	5	93.0 ± 3.2	94.8	90.7	93.2	92.9
IV	116	91	5	89.3 ± 3.1	90.1	88.2	92.1	87.2
**(d) NFC versus MBM-T**								
**Database**	**No. of NFC**	**No. of MBM-T**	**No. of PCs**	**Acc (%)**	**SE (%)**	**SP (%)**	**PPV (%)**	**NPV (%)**
I	169	152	2	96.5 ± 3.5	98.8	96.1	96.5	98.6
II	169	143	6	90.6 ± 2.9	92.9	94.4	95.2	91.8
III	115	73	5	94.1 ± 3.1	95.7	91.8	94.8	93.1
IV	116	72	5	93.6 ± 3.3	94	93.1	95.6	90.5

**Table 5:** Performance results of the LDA classifier in discriminating between different systems as pairs: (**a**) NFC versus (NIH/3T3 and MBM-T), (**b**) NFC versus NIH/3T3, (**c**) NFC versus MBM-T, and (**d**) NIH/3T3 versus MBM-T. The analysis was based on four wavenumber ranges, namely carbohydrates (1195–600 cm^−1^), proteins (amide III) and lipids (1380–1196 cm^−1^), nucleic acids (1520–1381 cm^−1^), and (**d**) proteins (amide I and II) (1728–1521 cm^−1^), of the Raman spectra of database I.

**(a) NFC versus (NIH/3T3 and MBM-T) (Normal–Abnormal)**								
**Region**	**No. of NFC**	**No. of (NIH/3T3 + MBM-T)**	**No. of PCs**	**Acc (%)**	**SE (%)**	**SP (%)**	**PPV (%)**	**NPV (%)**
1195–600	169	288	4	94.5	95.3	94.1	90.4	97.1
1380–1196			10	94.4	93.5	95.1	91.9	96.1
1520–1381			21	93	92.9	93.1	88.7	95.7
1728–1521			10	92.8	92.9	92.7	88.2	95.7
**(b) NIH/3T3 versus MBM-T (Cancerous–Precancerous)**								
	**No. of NFC**	**No. of NIH/3T3**	**No. of PCs**	**Acc (%)**	**SE (%)**	**SP (%)**	**PPV (%)**	**NPV (%)**
1195–600	136	152	32	72.2	67.6	76.3	71.9	72.5
1380–1196			12	72.9	64.7	80.3	74.6	71.8
1520–1381			8	67.4	61	73	66.9	67.7
1728–1521			11	71.9	69.1	74.3	70.7	72.9
**(c) NFC versus MBM-T (Normal–Cancerous)**								
	**No. of NFC**	**No. of MBM-T**	**No. of PCs**	**Acc (%)**	**SE (%)**	**SP (%)**	**PPV (%)**	**NPV (%)**
1195–600	169	152	11	97.8	97.6	98	98.2	97.4
1380–1196			8	96	97.6	94.1	94.8	97.3
1520–1381			10	95	95.9	94.1	94.7	95.3
1728–1521			6	95.3	96.4	94.1	94.8	96
**(d) NFC versus NIH/3T3 (Normal–Precancerous)**								
	**No. of NIH/3T3**	**No. of MBM-T**	**No. of PCs**	**Acc (%)**	**SE (%)**	**SP (%)**	**PPV (%)**	**NPV (%)**
1195–600	169	152	4	93.8	98.2	88.2	91.2	97.6
1380–1196			10	93.4	95.9	90.4	92.6	94.6
1520–1381			47	93.4	96.4	89.7	92.1	95.3
1728–1521			8	90.2	95.9	83.1	87.6	94.2

## Data Availability

All data and codes are available at https://github.com/MicrobiologyBiomedicalSpectroscopy/AerlyDetection.git (accessed on 3 July 2023).

## References

[1] Didkowska J., Wojciechowska U., Michalek I.M., Caetano dos Santos F.L. (2022). Cancer incidence and mortality in Poland in 2019. Sci. Rep..

[2] Wardle J., Robb K., Vernon S., Waller J. (2015). Screening for prevention and early diagnosis of cancer. Am. Psychol..

[3] Parsa N. (2012). Environmental factors inducing human cancers. Iran. J. Public Health.

[4] Baba A.I., Câtoi C. (2007). Comparative Oncology.

[5] Yancik R. (1997). Cancer burden in the aged: An epidemiologic and demographic overview. Cancer Interdiscip. Int. J. Am. Cancer Soc..

[6] Berger N.A., Savvides P., Koroukian S.M., Kahana E.F., Deimling G.T., Rose J.H., Bowman K.F., Miller R.H. (2006). Cancer in the elderly. Trans. Am. Clin. Climatol. Assoc..

[7] Hu Z., Tang J., Wang Z., Zhang K., Zhang L., Sun Q. (2018). Deep learning for image-based cancer detection and diagnosis—A survey. Pattern Recognit..

[8] Smith R.A., Cokkinides V., Eyre H.J. (2007). Cancer screening in the United States, 2007: A review of current guidelines, practices, and prospects. CA A Cancer J. Clin..

[9] Galway K., Black A., Cantwell M.M., Cardwell C.R., Mills M., Donnelly M. (2012). Psychosocial interventions to improve quality of life and emotional wellbeing for recently diagnosed cancer patients. Cochrane Database Syst. Rev..

[10] Salman A., Shufan E., Zeiri L., Huleihel M. (2013). Detection and identification of cancerous murine fibroblasts, transformed by murine sarcoma virus in culture, using Raman spectroscopy and advanced statistical methods. Biochim. Biophys. Acta.

[11] Das Gupta S., Finnilä M.A.J., Karhula S.S., Kauppinen S., Joukainen A., Kröger H., Korhonen R.K., Thambyah A., Rieppo L., Saarakkala S. (2020). Raman microspectroscopic analysis of the tissue-specific composition of the human osteochondral junction in osteoarthritis: A pilot study. Acta Biomater..

[12] Zhang F., Tan Y., Ding J., Cao D., Gong Y., Zhang Y., Yang J., Yin T. (2021). Application and Progress of Raman Spectroscopy in Male Reproductive System. Front. Cell Dev. Biol..

[13] Ibrahim O., Toner M., Flint S., Byrne H.J., Lyng F.M. (2021). The Potential of Raman Spectroscopy in the Diagnosis of Dysplastic and Malignant Oral Lesions. Cancers.

[14] Ralbovsky N.M., Lednev I.K. (2020). Towards development of a novel universal medical diagnostic method: Raman spectroscopy and machine learning. Chem. Soc. Rev..

[15] Huang Y., Swarup V.P., Bishnoi S.W. (2009). Rapid Raman imaging of stable, functionalized nanoshells in mammalian cell cultures. Nano Lett..

[16] Salman A., Shufan E., Zeiri L., Huleihel M. (2014). Characterization and detection of Vero cells infected with Herpes Simplex Virus type 1 using Raman spectroscopy and advanced statistical methods. Methods.

[17] Huleihel M., Shufan E., Zeiri L., Salman A. (2016). Detection of Vero Cells Infected with Herpes Simplex Types 1 and 2 and Varicella Zoster Viruses Using Raman Spectroscopy and Advanced Statistical Methods. PLoS ONE.

[18] Silveira L., Sathaiah S., Zângaro R.A., Pacheco M.T., Chavantes M.C., Pasqualucci C.A. (2003). Near-infrared Raman spectroscopy of human coronary arteries: Histopathological classification based on Mahalanobis distance. J. Clin. Laser Med. Surg..

[19] Ramos I.R.M., Malkin A., Lyng F.M. (2015). Current advances in the application of Raman spectroscopy for molecular diagnosis of cervical cancer. BioMed Res. Int..

[20] Rubina S., Krishna C.M. (2015). Raman spectroscopy in cervical cancers: An update. J. Cancer Res. Ther..

[21] Santos I.P., Caspers P.J., Bakker Schut T.C., van Doorn R., Noordhoek Hegt V., Koljenović S., Puppels G.J. (2016). Raman spectroscopic characterization of melanoma and benign melanocytic lesions suspected of melanoma using high-wavenumber Raman spectroscopy. Anal. Chem..

[22] Lui H., Zhao J., McLean D., Zeng H. (2012). Real-time Raman Spectroscopy for In Vivo Skin Cancer DiagnosisRaman Spectroscopy of Skin Cancer. Cancer Res..

[23] Haka A.S., Shafer-Peltier K.E., Fitzmaurice M., Crowe J., Dasari R.R., Feld M.S. (2005). Diagnosing breast cancer by using Raman spectroscopy. Proc. Natl. Acad. Sci. USA.

[24] Lazaro-Pacheco D., Shaaban A.M., Rehman S., Rehman I. (2020). Raman spectroscopy of breast cancer. Appl. Spectrosc. Rev..

[25] Hanna K., Krzoska E., Shaaban A.M., Muirhead D., Abu-Eid R., Speirs V. (2022). Raman spectroscopy: Current applications in breast cancer diagnosis, challenges and future prospects. Br. J. Cancer.

[26] Zheng Q., Li J., Yang L., Zheng B., Wang J., Lv N., Luo J., Martin F.L., Liu D., He J. (2020). Raman spectroscopy as a potential diagnostic tool to analyse biochemical alterations in lung cancer. Analyst.

[27] Karnachoriti M., Stathopoulos I., Kouri M., Spyratou E., Orfanoudakis S., Lykidis D., Lambropoulou Μ., Danias N., Arkadopoulos N., Efstathopoulos E. (2023). Biochemical differentiation between cancerous and normal human colorectal tissues by micro-Raman spectroscopy. Spectrochim. Acta Part A Mol. Biomol. Spectrosc..

[28] Khan M., Gasser S. (2016). Generating Primary Fibroblast Cultures from Mouse Ear and Tail Tissues. J. Vis. Exp..

[29] Berman J.J., Albores-Saavedra J., Bostwick D., DeLellis R., Eble J., Hamilton S.R., Hruban R.H., Mutter G.L., Page D., Rohan T. (2006). Precancer: A conceptual working definition: Results of a Consensus Conference. Cancer Detect. Prev..

[30] Marim F.M., Silveira T.N., Lima Jr D.S., Zamboni D.S. (2010). A method for generation of bone marrow-derived macrophages from cryopreserved mouse bone marrow cells. PLoS ONE.

[31] Stone N., Kendall C., Smith J., Crow P., Barr H. (2004). Raman spectroscopy for identification of epithelial cancers. Faraday Discuss..

[32] Auner G.W., Koya S.K., Huang C., Broadbent B., Trexler M., Auner Z., Elias A., Mehne K.C., Brusatori M.A. (2018). Applications of Raman spectroscopy in cancer diagnosis. Cancer Metastasis Rev..

[33] Schulz H., Baranska M. (2007). Identification and quantification of valuable plant substances by IR and Raman spectroscopy. Vib. Spectrosc..

[34] Teh S.K., Zheng W., Ho K.Y., Teh M., Yeoh K.G., Huang Z. (2010). Near-infrared Raman spectroscopy for optical diagnosis in the stomach: Identification of helicobacter-pylori infection and intestinal metaplasia. Int. J. Cancer.

[35] Stone N., Kendall C., Shepherd N., Crow P., Barr H. (2002). Near-infrared Raman spectroscopy for the classification of epithelial pre-cancers and cancers. J. Raman Spectrosc..

[36] Chan J.W., Taylor D.S., Zwerdling T., Lane S.M., Ihara K., Huser T. (2006). Micro-Raman spectroscopy detects individual neoplastic and normal hematopoietic cells. Biophys. J..

[37] Cheng W.T., Liu M.T., Liu H.N., Lin S.Y. (2005). Micro-Raman spectroscopy used to identify and grade human skin pilomatrixoma. Microsc. Res. Tech..

[38] Gaeta R., Franchi A., Capanna R., Mario D.A. (2019). Contribution of Raman Spectroscopy to diagnosis and grading of chondrogenic tumours. Virchows Arch..

[39] Ye J., Tian Z., Wei H., Li Y. (2020). Baseline correction method based on improved asymmetrically reweighted penalized least squares for the Raman spectrum. Appl. Opt..

[40] Bishop C.M., Nasrabadi N.M. (2006). Pattern Recognition and Machine Learning.

[41] Duda R.O., Hart P.E., Stork D.G. (2001). Pattern Classification.

[42] Salman A., Lapidot I., Pomerantz A., Tsror L., Shufan E., Moreh R., Mordechai S., Huleihel M. (2012). Identification of fungal phytopathogens using Fourier transform infrared-attenuated total reflection spectroscopy and advanced statistical methods. J. Biomed. Opt..

[43] Hastie T., Tibshirani R., Friedman J.H., Friedman J.H. (2009). The Elements of Statistical Learning: Data Mining, Inference, and Prediction.

[44] Subasi A., Gursoy M.I. (2010). EEG signal classification using PCA, ICA, LDA and support vector machines. Expert Syst. Appl..

[45] Pardee A.B. (1976). Cancer Cells and Normal Cells. Proc. Am. Philos. Soc..

[46] Stowell S.R., Ju T., Cummings R.D. (2015). Protein glycosylation in cancer. Annu. Rev. Pathol..

[47] Levine A.J., Puzio-Kuter A.M. (2010). The control of the metabolic switch in cancers by oncogenes and tumor suppressor genes. Science.

[48] Fadaka A., Ajiboye B., Ojo O., Adewale O., Olayide I., Emuowhochere R. (2017). Biology of glucose metabolization in cancer cells. J. Oncol. Sci..

